# Complement Factor H Inhibits Anti-Neutrophil Cytoplasmic Autoantibody-Induced Neutrophil Activation by Interacting With Neutrophils

**DOI:** 10.3389/fimmu.2018.00559

**Published:** 2018-03-19

**Authors:** Su-Fang Chen, Feng-Mei Wang, Zhi-Ying Li, Feng Yu, Min Chen, Ming-Hui Zhao

**Affiliations:** ^1^Renal Division, Department of Medicine, Peking University First Hospital, Institute of Nephrology, Peking University, Beijing, China; ^2^Key Laboratory of Renal Disease, Ministry of Health of China, Beijing, China; ^3^Key Laboratory of Chronic Kidney Disease Prevention and Treatment, Peking University, Ministry of Education, Beijing, China; ^4^Peking-Tsinghua Center for Life Sciences, Beijing, China

**Keywords:** factor H, neutrophils, activation, anti-neutrophil cytoplasmic autoantibody, vasculitis

## Abstract

Our previous study demonstrated that plasma levels of complement factor H (FH) were inversely associated with the disease activity of patients with anti-neutrophil cytoplasmic autoantibody (ANCA)-associated vasculitis (AAV). In addition to serving as an inhibitor of the alternative complement pathway, there is increasing evidence demonstrating direct regulatory roles of FH on several cell types. Here, we investigated the role of FH in the process of ANCA-mediated activation of neutrophils and neutrophil–endothelium interaction. We demonstrated that FH bound to neutrophils by immunostaining and flow cytometry. Interestingly, ANCA-induced activation of neutrophils, including respiratory burst and degranulation, was inhibited by FH. Although FH enhanced neutrophils adhesion and migration toward human glomerular endothelial cells (hGEnCs), it inhibited ANCA-induced activation of neutrophils in the coculture system of hGEnCs and neutrophils. Moreover, the activation and injury of hGEnCs, reflected by the level of endothelin-1 in the supernatant of cocultures, was markedly reduced by FH. However, we found that FH from patients with active AAV exhibited a deficient ability in binding neutrophils and inhibiting ANCA-induced neutrophil activation in fluid phase and on endothelial cells, as compared with that from healthy controls. Therefore, our findings indicate a novel role of FH in inhibiting ANCA-induced neutrophil activation and protecting against glomerular endothelial injury. However, FH from patients with active AAV are deficient in their ability to bind neutrophils and inhibit neutrophil activation by ANCA. It further extends the current understanding of the pathogenesis of AAV, thus providing potential clues for intervention strategies.

## Introduction

Anti-neutrophil cytoplasmic autoantibody (ANCA)-associated vasculitis (AAV) is a group of potentially life-threatening autoimmune diseases, characterized by pauci-immune necrotizing vasculitis of small vessels and circulating autoantibodies targeting the cytoplasmic constituents of neutrophils, especially proteinase 3 (PR3) and myeloperoxidase (MPO) (1). It includes granulomatosis with polyangiitis, microscopic polyangiitis, and eosinophilic granulomatosis with polyangiits (2). ANCA-induced neutrophil activation is crucial for the development of AAV. Using the mouse model of ANCA-associated crescentic glomerulonephritis induced by anti-MPO IgG, Xiao et al. demonstrated that ANCA and neutrophils are indispensable for the initiation of necrotizing crescentic glomerulonephritis (3, 4). *In vitro*, ANCAs are capable of inducing activation of primed neutrophils, resulting in respiratory burst and degranulation of neutrophils, which may directly participate in the development of vascular inflammation (5–8). Moreover, “endothelium–neutrophil interactions are essential to allow neutrophils to move toward inflammatory sites” and trigger an explosive activation of neutrophils by ANCA (9). In the past decade, accumulating evidence has demonstrated that activation of complement system *via* the alternative pathway is crucial for the development of AAV (10–15). Activation of neutrophils induced by ANCA results in releasing factors that trigger the activation of the alternative complement pathway and subsequently generating C5a (12). C5a further primes neutrophils for activation by ANCA (12, 16, 17), thus causing a self-amplification loop for ANCA-induced neutrophil activation.

Complement factor H (FH) is an abundant plasma glycoprotein that functions as a key regulator of the alternative complement activation by accelerating the decay of the C3 convertase (C3bBb) and by acting as a cofactor for factor I-mediated cleavage of C3b (18, 19). Our recent study found that plasma levels of FH were inversely associated with the disease activity and renal damage of AAV patients (20); and, an impaired complement regulatory activity of FH was found in AAV patients (21), indicating an important role of FH in the disease development. In addition to serving as an inhibitor of the alternative complement pathway, there is increasing evidence demonstrating direct regulatory roles of FH on several cell types. Mihlan et al. showed that FH inhibited the production of pro-inflammatory cytokines by activated macrophages during phagocytosis (22). As for neutrophils, “FH has been shown to bind to neutrophils *via* complement receptor type 3” (CR3; α_Mβ2_ integrin; CD11b/CD18) (23–25), and mediate adhesion and migration of neutrophils by serving as an adhesion molecule for neutrophils (24–26). Losse et al. further found that *Candida albicans*-bound FH facilitated neutrophil antimicrobial activity by enhancing the recognition of fungal (25). Given that CD11b/CD18, as the ligand for FH binding on neutrophils, plays an indispensable role in ANCA-mediated activation of neutrophils and leukocyte–endothelium interactions in the presence of anti-MPO antibodies (27, 28), we hypothesized that FH may participate in the process of ANCA-mediated activation of neutrophils and neutrophil–endothelium interaction, thus influencing the amplification loop between activation of neutrophils and the alternative complement pathway.

## Materials and Methods

### Patients

Twelve patients who were newly diagnosed with active AAV in the Department of Nephrology, Peking University First Hospital from 2013 to 2014 were enrolled in this study. All these patients met the definition of AAV according to “the 2012 revised International Chapel Hill Consensus Conference Nomenclature of Vasculitides” (2). All the patients received plasma exchange therapy. Plasma samples of these patients were obtained at the beginning of the plasma exchange process prior to initiating the infusion of blood products and immunosuppressive therapy. Plasma samples were stored at −80°C in small aliquots until use. Four age-matched healthy blood donors were included as normal controls. Informed consent was obtained from each participant. The study was in compliance with the Declaration of Helsinki and was approved by the ethics committees of Peking University First Hospital.

### Cells

Human neutrophils from healthy donors were isolated from anticoagulated peripheral blood by density gradient centrifugation using Polymorphprep and Lymphoprep (Nycomed, Oslo, Norway). Erythrocytes were lyzed by erythrocytes lyzing buffer (Tiangen Biotech, Beijing, China). Then, neutrophils were washed in Ca^2+^/Mg^2+^ free Hanks balanced salt solution (HBSS^−/−^) (Gibco, Grand Island, NY, USA) and prepared for further analysis.

Human glomerular endothelial cells (hGEnCs) (ScienCell Research Laboratories, San Diego, CA, USA) were grown at 37°C and 5% CO_2_ in endothelial cell medium (ECM) (ScienCell Research Laboratories, San Diego, CA, USA) supplemented with 10% fetal bovine serum (FBS), 1% penicillin/streptomycin solution, and 1% endothelial cell growth factor, according to the manufacturer’s instructions. All experiments were performed using hGEnCs at passages 3–5. For the synchronization of cell cycle, hGEnCs were kept in serum-free ECM without endothelial cell growth supplement for 12 h prior to experiments without bio coating.

### Preparation of IgG

Anti-neutrophil cytoplasmic autoantibody-positive IgG was isolated from plasma of AAV patients with positive ANCA by affinity chromatography using a HiTrap Protein G HP column in an AKTA-FPLC system (GE Healthcare, Chicago, IL, USA) according to the methods described previously (29, 30).

### Purification of FH From Plasma

Factor H was isolated from plasma of 12 patients with active AAV by three sequential chromatographic columns, consisting of l-lysine Sepharose 4B column, Resource Q column, and Superdex 200 high resolution HiLoad 16/600 column (GE Healthcare, Chicago, IL, USA), as described previously (21). FH from four age-matched healthy volunteers were also purified as normal controls. The purity of the final preparations of FH was determined to be comparable to commercial purified FH (Calbiochem, Darmstadt, Germany) by SDS-PAGE (see Figure S1 in Supplementary Material), which is consistent with previous studies (21, 31).

### Binding of FH to Neutrophils

Binding of FH to neutrophils was analyzed by immunofluorescence staining and flow cytometry according to previously described methods (25, 26), with some minor modifications. Briefly, neutrophils isolated from healthy blood donors were seeded on eight-well chamber slides in Roswell Park Memorial Institute (RPMI) 1640 supplemented with 0.5% FBS at a density of 10^5^ cells/well and allowed to adhere for 1 h at 37°C. Then, neutrophils were incubated with 50 µg/ml FH or medium followed by fixed with 4% paraformaldehyde. After blocking, bound FH were detected using a goat polyclonal antibody directed against human FH (Calbiochem, Darmstadt, Germany) followed by Alexa Fluor 488-conjugated donkey anti-goat IgG (Molecular Probes-Invitrogen, Carlsbad, CA, USA). Normal goat IgG (Calbiochem, Darmstadt, Germany) was applied as the isotype control for the primary staining antibody. After washing, slides were mounted in fluorescence preserving medium fortified with 4′, 6-diamidino-2-phenylindole (ZSGB-BIO, Beijing, China). Immunofluorescence staining was visualized using a confocal microscope (Carl Zeiss, Germany).

For flow cytometry analysis, prepared neutrophils were suspended in “modified Hank’s buffer (142 mM NaCl, 1 mM Na_2_SO_4_, 5 mM KCl, 1 mM NaH_2_PO_4_, 1 mM MgCl_2_, 2.5 mM CaCl_2_, 5 mM glucose, 10 mM HEPES; pH 7.4)” (26) containing 1% BSA to a concentration of 1 × 10^6^ cells/ml. After blocking, cells were incubated with 100 µg/ml FH for 30 min at 30°C with gentle shaking. All further steps were performed in PBS containing 1% BSA at 4°C. After washing, a goat anti-human FH antibody was added for 30 min, followed by Alexa488-conjugated donkey anti-goat IgG for 30 min. Neutrophils were analyzed using a FACScan (Becton Dickinson, Heidelberg, Germany). Neutrophils were identified by forward/sideward scatter (FSC/SSC) and data were collected from 10,000 cells per sample.

### Analysis of Activate CR3 (CD11b/CD18)

The expression level of active CR3 on neutrophils was detected according to previously described method (32), with some minor modifications. Briefly, prepared neutrophils were suspended in RPMI 1640 to a concentration of 2.5 × 10^6^ cells/ml. Then 100 µg/ml FH, 5 ng/ml TNF-α (Sigma, St. Louis, MO, USA), or 1 × 10^−8^M fMLP (Sigma, St. Louis, MO, USA) were added, respectively. Unstimulated cells incubated with medium were set as the blank controls. Thereafter, cells were incubated at 37°C for 30 min. After washing, cells were incubated with phycoerythrin-conjugated anti-CD11b monoclonal antibodies CBRM1/5 (eBioscience, San Diego, CA, USA) or isotype-matched control antibodies at 4°C for 30 min. Neutrophils were analyzed using a FACScan instrument. Neutrophils were gated by FSC and SSC, and 10,000 cells per sample were routinely collected for data analysis.

### Adhesion Assays

To investigate the effect of FH on the interaction between neutrophils and endothelial cells, adhesion assays were performed. Endothelial monolayers were grown on wells of Costar 96-well black transparent-bottom plates (Corning Life Sciences, Corning, NY, USA) and pretreated with 50 µg/ml FH, human serum albumin (HSA), or medium for 1 h at 37°C. Prepared neutrophils were suspended in RPMI 1640 medium without serum to a concentration of 1 × 10^6^ cells/ml and then stained with 5 µM Cell tracker green (Invitrogen) for 45 min at 37°C. After washing, neutrophils in serum-free ECM were primed with 2 ng/ml TNF-α and then added to the wells to adhere for 1 h at 37°C. In some experiments, TNF-α primed neutrophils were pre-incubated with 100 µg/ml FH or controls for 1 h at 37°C, followed by added to non-treated hGEnC monolayers. Non-adherent cells were removed by extensive washing. Adherent cells were reflected by fluorescence intensity (FI) of neutrophils measured by a fluorescence reader (TriStar Multimode Microplate Reader LB941, Berthold Technologies, Bad Wildbad, Germany) with filters of 495 nm (excitation) and 515 nm (emission).

### Neutrophil Migration Assays

Neutrophil migration assays were performed using 24-well Costar transwell plates with 3 μm-pore polycarbonate membranes inserts (Corning Life Sciences, Corning, NY, USA) according to the previously described method (25, 26), with some modifications. Endothelial monolayers grown on the lower chamber were pre-incubated with 50 µg/ml FH, HSA, or serum-free ECM medium. Neutrophils were stained with 5 µM Cell tracker green for 45 min at 37°C. After washing, 10^6^ neutrophils in serum-free ECM were added to the top chamber for 60 min at 37°C. “Then 25 mM EDTA was added to the lower chamber to release neutrophils adhering to the bottom of the membrane and the bottom of the well” (25). Migrated neutrophils were reflected by FI measured using a fluorescence reader with filters of 495 nm (excitation) and 515 nm (emission).

### Measurement of Reactive Oxygen Species and Lactoferrin Released From Neutrophils

The generation of reactive oxygen species (ROS) by ANCA stimulated neutrophils was assessed using dihydrorhodamine (Sigma-Aldrich, St. Louis, MO, USA) as previously described (16). Neutrophils (2.5 × 10^6^/ml) were pre-incubated with 5 µg/ml dihydrorhodamine in HBSS^−/−^ for 30 min at 37°C. After washing, neutrophils were primed with 5 ng/ml TNF-α in modified Hank’s buffer for 15 min, followed by incubation with 100 µg/ml FH or buffer for 30 min at 37°C. Then neutrophils were stimulated by ANCA-positive IgGs for 1 h. The reaction was stopped by centrifugation and suspension with 1 ml of ice-cold HBSS containing 1% BSA. Cells were analyzed using a FACScan. Neutrophils were gated by FSC and SSC, and 10,000 cells per sample were routinely collected for data analysis. The amount of generated reactive oxygen was represented by the mean fluorescence intensity (MFI).

To detect the generation of ROS by neutrophils cocultured with hGEnCs. Neutrophils pre-incubated with FH or HSA were added to endothelial monolayers grown on Costar 96-well black transparent-bottom plates and allowed to adhere for 1 h, followed by stimulation with ANCA-positive IgGs for 2 h. The fluorescence signal of the oxidized dihydrorhodamine was measured using a fluorescence reader with excitation and emission filter settings of 485 and 535 nm, respectively.

For detection of lactoferrin, which is considered as a biomarker of neutrophil degranulation, supernatants of neutrophils or cocultures were collected. Supernatant levels of lactoferrin were tested by ELISA using a commercial kit following the instruction provided by the manufacturer (Abcam, Cambridge, MA, USA).

### Evaluation of Endothelium Injury by Endothelin-1 (ET-1) Quantification

Endothelin-1 was considered as a biomarker of endothelial cell activation and injury (33), and neutrophils cannot release ET-1. Therefore, supernatants of the coculture system were collected for ET-1 measurement using a commercial ELISA kit (R&D Systems, Minneapolis, MN, USA), following the instruction provided by the manufacturer.

### Mass Spectrometry Analysis of FH

Factor H purified from patients with active AAV and healthy controls, as well as commercially derived FH were further analyzed by mass spectrometry for detection of possible modifications. Samples were incubated in 8 M urea, 10 mM DTT in 50 mM ammonium bicarbonate (NH_4_HCO_3_) at 56°C for 40 min, followed by incubating in 55 mM iodoacetamide in 50 mM NH_4_HCO_3_ at room temperature for 1 h. The solution was then diluted with 50 mM NH_4_HCO_3_ to a final urea concentration of 2 M and digested with trypsin in 50 mM NH_4_HCO_3_ overnight at 37°C. The resulting peptides were centrifuged to dryness in vacuum. For LC-MS/MS analysis, the samples were re-dissolved in 0.2% formic acid and separated on a 75 μm × 15 cm capillary column packed with 4 µm C18 bulk material (InnosepBio, China) using an nLC 1000 system (Thermo Scientific, USA). Peptides were eluted in a linear gradient consisting of mobile phases A and B, which were 0.1% formic acid in water and 0.1% formic acid in acetonitrile, respectively. The elution system started with a linear gradient from 5% B to 30% B in 60 min, followed by 30% B to 75% B in the next 4 min, and maintained at 75% B for another 20 min. The eluted peptides were then sprayed directly into a Velos Pro Orbitrap Elite mass spectrometer (Thermo Scientific, USA) equipped with a nano-ESI source. The mass spectrometer was run in data-dependent mode with a full MS scan resolution of 120,000 in FT mode followed by collision-induced dissociation MS/MS scans of the 15 most abundant ions in the initial MS scan.

Database search were carried out using Mascot (version 2.3.02) for detecting variable modifications including Carbamidomethyl (Cys), Oxidation (Met/Cys), Nitration (Tyr), and Chlorination (Tyr). The mass tolerance was set to be ±10 ppm for peptide pass tolerance and ±0.6 Da for fragment mass tolerance.

### Statistical Analysis

Quantitative data were presented as mean ± SD or median and interquartile range, according to the normality of data. The between-group differences in normally distributed quantitative parameters were assessed by *t* test or ANOVA as appropriate. The difference was considered statistically significant when the *P* values < 0.05. Analysis was performed with SPSS statistical software package (version 13.0, Chicago, IL, USA).

## Results

### Clinical Data of Patients With Active AAV

Of the 12 patients with active AAV, seven were male and five were female, with an average age of 61.9 ± 8.4 (range 48–74) years at diagnosis. All the patients were ANCA positive, 10 patients were positive for MPO-ANCA and 2 were positive for PR3-ANCA. At the time of diagnosis, the level of serum creatinine was 634.9 ± 260.0 (range 254.2–1,118.0) μmol/l; the level of Birmingham Vasculitis Activity Scores was 20.8 ± 4.4 (range 12–29).

### Binding of FH to Neutrophils

**Figure 1:**
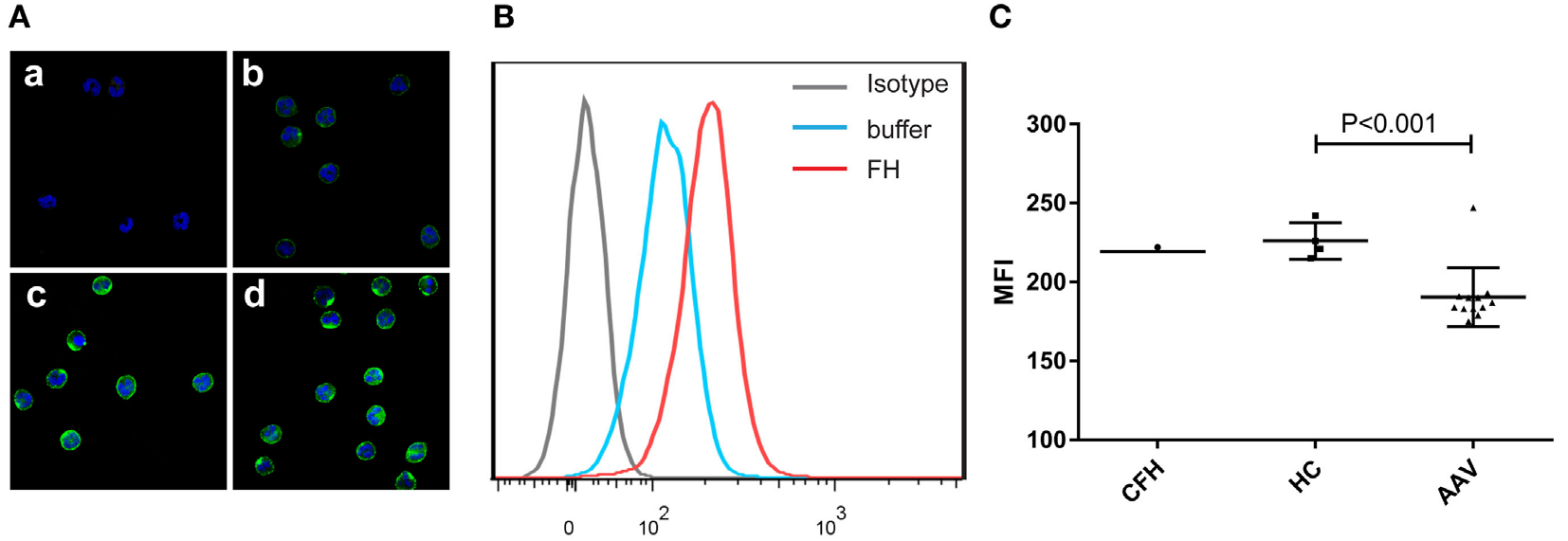
Binding of factor H (FH) to human neutrophils. Neutrophils were incubated with FH or buffer, and bound FH was detected using goat anti-human FH antibody. Normal goat IgG was using as the relevant isotype control antibody. **(A)** Representative confocal images show binding of FH to the cell surface of neutrophils. [**(A)**, a] Isotype control; [**(A)**, b] negative control performed by incubating neutrophils with buffer only; [**(A)**, c,d] neutrophils were incubation with FH followed by detection using goat anti-human FH antibody. **(B)** Shown is a representative histogram out of three independent experiments performed by flow cytometry. **(C)** Flow cytometry analysis of binding of FH to neutrophils. FH purified from healthy controls exhibited similar binding activity to commercial FH, while FH from patients with active ANCA-associated vasculitis bound less efficiently to neutrophils.

Previous studies reported that FH bound to neutrophils and influenced the activation of neutrophils upon certain stimulation (25, 26). Therefore, we analyzed the binding of FH to neutrophils. FH showed specific binding to human neutrophils by immunostaining and flow cytometry (Figures 1A,B), which is consistent with previous findings (23–26). FH purified from healthy controls exhibited similar binding activity to commercially derived FH. However, FH from patients with active AAV bound less efficiently to neutrophils, as compared with that from normal controls (189.6 ± 15.8 vs. 226.0 ± 11.6, *P* = 0.001) (Figure 1C).

### Binding of FH to Neutrophils Do Not Activate CR3

**Figure 2:**
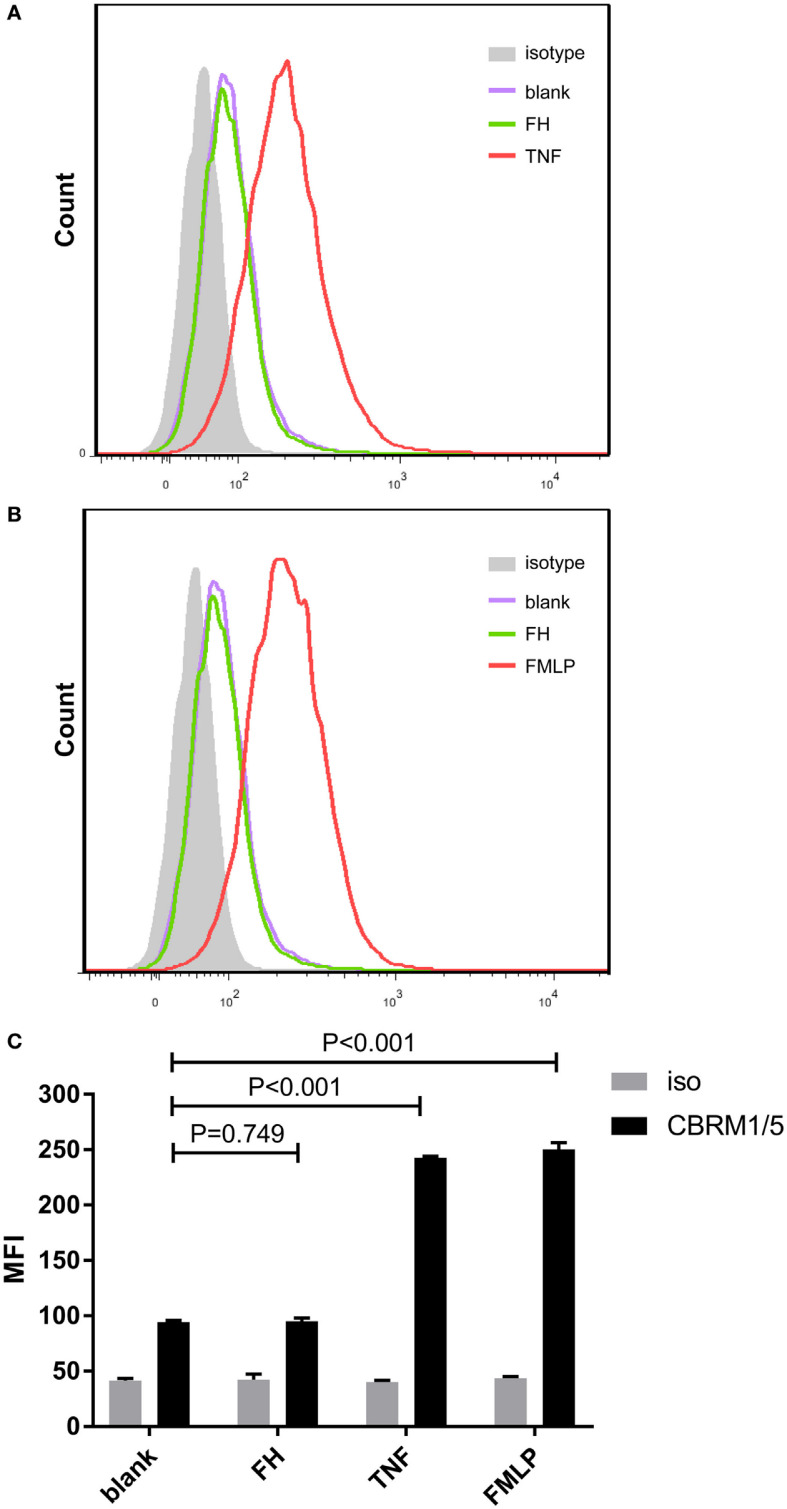
Binding of factor H (FH) to neutrophils do not activate CR3 (CD11b/CD18). Activate CR3 on neutrophils were detected using phycoerythrin-conjugated anti-CD11b monoclonal antibodies CBRM1/5 after incubation with FH, TNF-α, or fMLP. Unstimulated cells incubated with medium were set as blank controls. **(A,B)** Representative histograms showing that priming neutrophils with TNF-α or fMLP-induced expression of activate CR3, while FH did not activate CR3. **(C)** Analysis of expression of activate CR3. The expression level of active CR3 on nuetrophils after incubation with FH was comparable to unstimulated neutrophils. The values represent mean ± SD derived from three independent experiments.

Previous studies demonstrated that CR3 (α_Mβ2_ integrin; CD11b/CD18) serves as a specific receptor for FH on neutrophils (24–26). Therefore, to investigate whether CR3 is activated with FH incubation, a monoclonal antibody (CBRM1/5) which specifically directs against the active domain of CD11b was used to detect the expression level of active CR3. Consistent with previous studies, a low-level activation of CR3 was observed at baseline, while priming neutrophils with TNF-α or fMLP significantly increased the expression of active CR3 (32, 34). Incubation of neutrophils with FH did not activate CR3. When incubating neutrophils with FH, the expression level of active CR3 on neutrophils was comparable to that of unstimulated cells (94.3 ± 1.5 vs. 95.0 ± 3.0, *P* = 0.749) (Figure 2).

### FH Inhibits ANCA-Induced Respiratory Burst and Degranulation of Neutrophils

**Figure 3:**
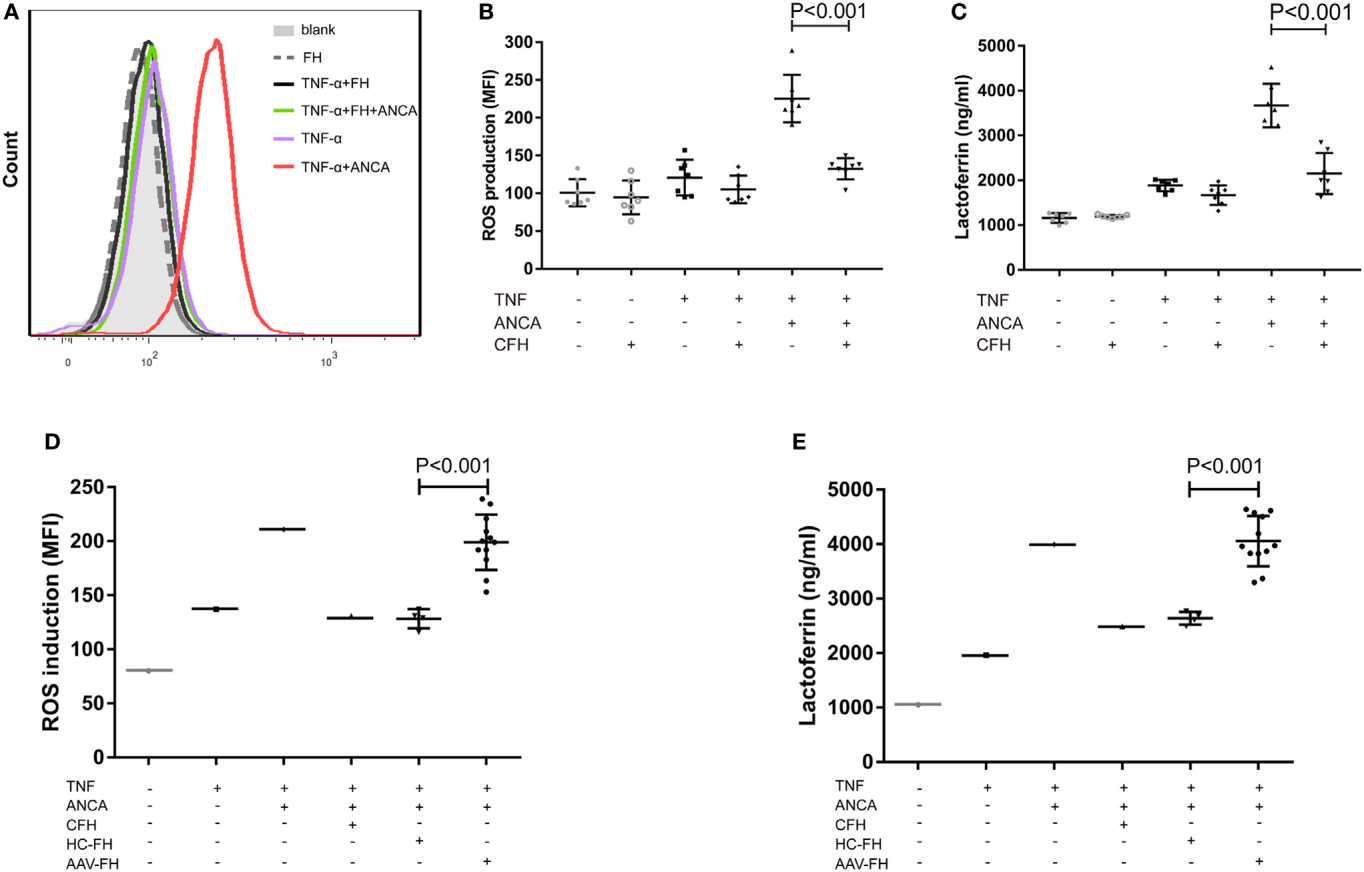
Factor H (FH) inhibits anti-neutrophil cytoplasmic autoantibody (ANCA)-induced respiratory burst and degranulation of neutrophils. **(A,B)** Neutrophils were primed with TNF-α followed by activation with myeloperoxidase-ANCA positive IgGs or proteinase 3-ANCA positive IgGs. Pre-incubation of neutrophils with FH resulted in marked reduction of generation of reactive oxygen species (ROS). **(A)** Shown is a representative histogram out of seven independent experiments. **(B)** The bars and error bars represent mean ± SD of ROS production in seven independent experiments using ANCA-IgGs derived from different patients with ANCA-associated vasculitis (AAV). **(C)** Neutrophils pre-incubated with FH showed less degranulation of lactoferrin. The values represent mean ± SD derived from seven independent experiments. **(D,E)** FH from patients with active AAV exhibited deficient ability in inhibiting respiratory burst and degranulation of neutrophils activated by ANCA, as compared with FH from healthy controls. Shown is mean ± SD of ROS and lactoferrin production by neutrophils pre-incubated with FH from different patients or controls, respectively. Experiments were independently repeated three times.

Anti-neutrophil cytoplasmic autoantibody-mediated activation of neutrophils plays a central role in the pathogenesis of AAV. Therefore, to further investigate whether FH influences ANCA-induced activation of neutrophils, human neutrophils were primed with TNF-α followed by activation with ANCA-positive IgGs. When neutrophils were pre-incubated with FH before stimulated by ANCA, the level of respiratory burst of neutrophils was significantly decreased, as determined by the MFI representing the intracellular development of ROS in neutrophils (132.4 ± 14.2 vs. 225.4 ± 31.4, *P* < 0.001). The level of ROS generation by neutrophils pre-incubated with FH was similar to that of neutrophils merely primed with TNF-α (Figures 3A,B). Degranulation of neutrophils was evaluated by measuring lactoferrin level in the supernatant of neutrophils in parallel. Consistently, pre-incubation of neutrophils with FH resulted in significantly less lactoferrin release from neutrophils upon activation by ANCA-positive IgGs (2,152.1 ± 459.1 ng/ml vs. 3,669.2 ± 487.1 ng/ml, *P* < 0.001) (Figure 3C).

Then we investigated the effect of FH from patients with AAV on ANCA-mediated activation of neutrophils. We found that FH from normal controls inhibited ANCA-induced activation of neutrophils, comparable to commercially derived FH. However, under the same condition, FH from active AAV patients could not effectively inhibit neutrophil activation upon ANCA stimulation, including ROS generation and lactoferrin degranulation, as compared with those from normal controls (199.1 ± 25.6 vs. 128.3 ± 8.8, *P* < 0.001; 4,055.6 ± 462.4 ng/ml vs. 2,639.6 ± 117.1 ng/ml, *P* < 0.001, respectively). The mean levels were similar to that of neutrophils not treated with FH prior to activation by ANCA (Figures 3D,E).

### FH Enhances Neutrophils Adhesion and Migration Toward hGEnCs

**Figure 4:**
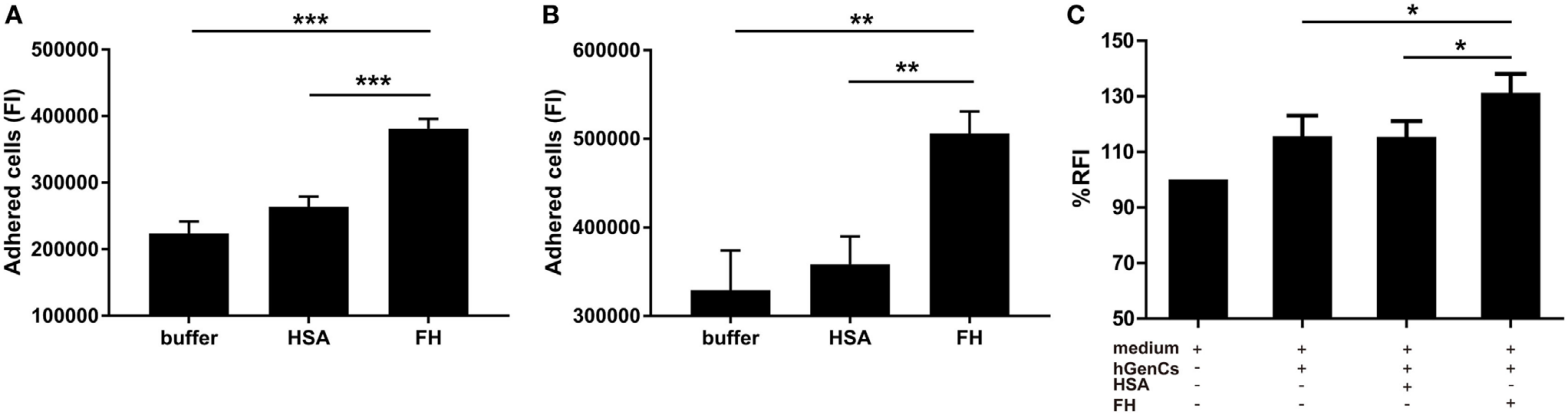
Factor H (FH) enhances neutrophil adhesion and migration to hGEnCs. In all assays, neutrophils were stained with Cell tracker green. **(A)** TNF-α primed neutrophils pre-incubated with FH or controls were added to endothelial monolayers and allowed to adhere. Adherent cells were quantified by plate fluorimeter. **(B)** hGEnC monolayers were pretreated with FH or controls. TNF-α primed neutrophils were added and allowed to adhere. Adherent cells were quantified. Results are expressed as mean ± SD of fluorescence intensity (FI) values derived from five independent experiments. **(C)** hGEnC monolayers grown on the lower chamber of transwells were pre-incubated with FH, human serum albumin (HSA), or medium. TNF-α primed neutrophils were added to the upper wells. The migration of neutrophils through a membrane insert was quantified by measuring the fluorescence in the lower chamber. Transwells without cultivated hGEnCs were used as negative controls. The data were normalized after subtraction of negative controls and represent mean percentage values ± SD from five experiments performed with different cell donors (*P < 0.05; **P < 0.01; ***P < 0.001).

Factor H was previously suggested as an adhesion molecule for human neutrophils (24), therefore we hypothesized that FH may facilitate the interaction between neutrophils and endothelial cells by interacting with glycosaminoglycans. To test this hypothesis, we performed adhesion and migration assays. We found that pre-incubation of TNF-α primed neutrophils with FH resulted in significantly increased number of neutrophils adhered to hGEnCs, as compared with pre-incubation with buffer or HSA (38.1 × 10^4^ ± 5.1 × 10^4^ vs. 22.4 × 10^4^ ± 4.7 × 10^4^, *P* < 0.001; 38.1 × 10^4^ ± 5.1 × 10^4^ vs. 26.4 × 10^4^ ± 4.1 × 10^4^, *P* < 0.001, respectively). When endothelial cells were pretreated with FH before addition of primed neutrophils, adhesion of neutrophils were also enhanced (50.6 × 10^4^ ± 5.5 × 10^4^ vs. 32.9 × 10^4^ ± 10.0 × 10^4^, *P* = 0.009; 50.6 × 10^4^ ± 5.5 × 10^4^ vs. 35.8 × 10^4^ ± 7.1 × 10^4^, *P* = 0.006, respectively) (Figures 4A,B). Consistently, pre-incubation of endothelial cells on the lower chamber of transwells with FH resulted in increased number of neutrophils migrated through the membrane inserts, as compared with HSA or medium (131.3 ± 6.7 vs. 115.5 ± 5.7%, *P* = 0.011; 131.3 ± 6.7 vs. 115.6 ± 6.1%, *P* = 0.013, respectively) (Figure 4C). Collectively, these data provides evidence to support the hypothesis that FH may facilitate the interaction between neutrophils and endothelial cells by serving as an adhesion molecule for neutrophils.

### FH Inhibits ANCA-Induced Neutrophil Activation and Endothelial Injury in the Coculture System of hGEnCs and Neutrophils

**Figure 5:**
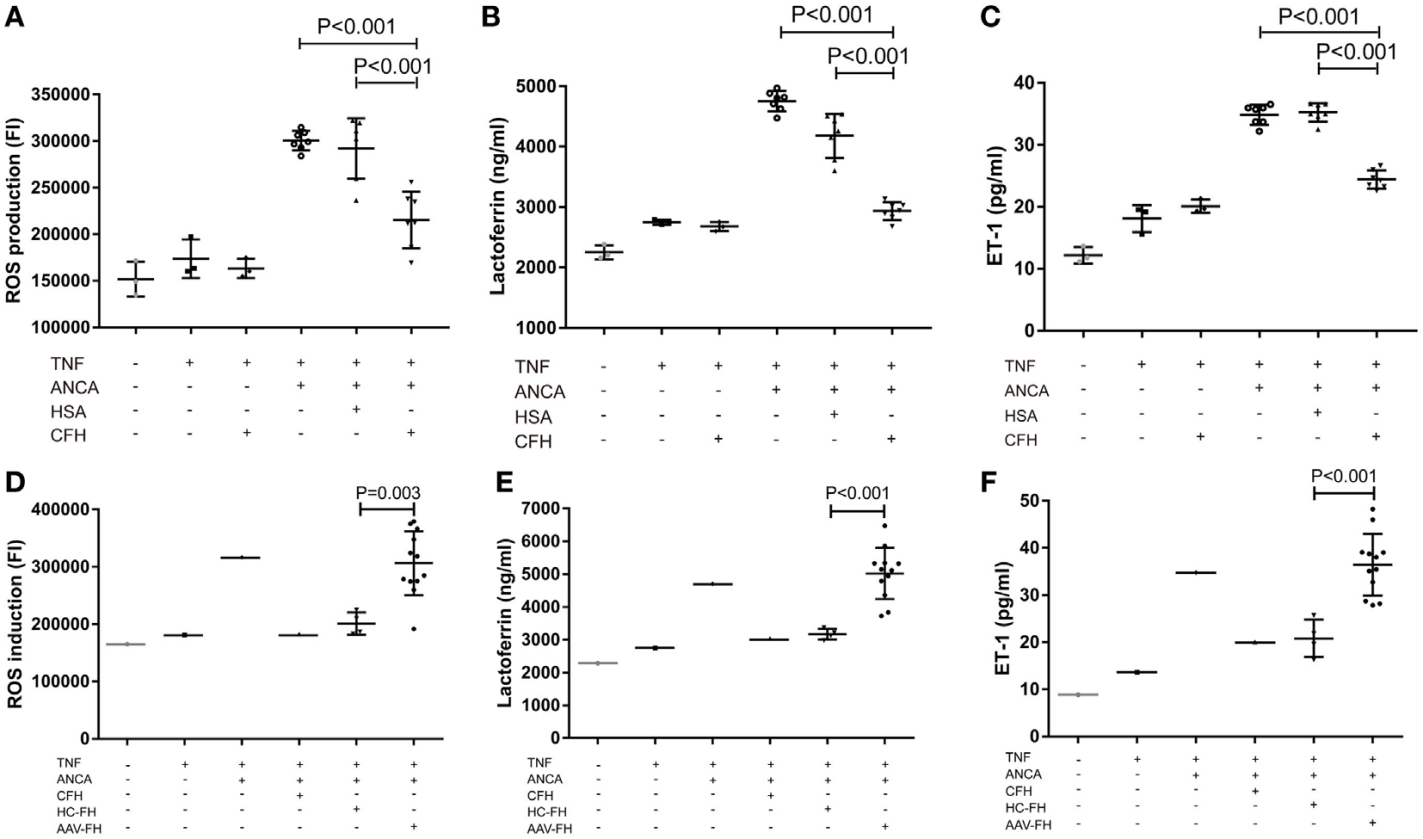
Factor H (FH) inhibits anti-neutrophil cytoplasmic autoantibody (ANCA)-induced neutrophil activation and endothelial injury in the coculture system of hGEnCs and neutrophils. Neutrophils primed with TNF-α were allowed to adhere on endothelial monolayers and followed by activation with myeloperoxidase-ANCA positive IgGs or proteinase 3-ANCA positive IgGs. **(A)** Pre-incubation of neutrophils with FH resulted in less production of ROS in the cocultures of hGEnCs and neutrophils. The values represent mean ± SD of ROS production derived from three independent experiments using ANCA-IgGs from seven different patients with ANCA-associated vasculitis (AAV). **(B)** The level of lactoferrin in the coculture supernatants was decreased by pre-incubation of neutrophils with FH. Shown is mean ± SD of three independent experiments. **(C)** The levels of endothelin-1 (ET-1) in the supernatant of cocultures decreased significantly when pre-incubating neutrophils with FH. **(D,E)** FH from patients with active AAV exhibited deficient ability in inhibiting the production of ROS and degranulation of lactoferrin by neutrophils cocultured with hGEnCs in the presence of ANCA, as compared with FH from healthy controls. Shown is mean ± SD of ROS and lactoferrin production by neutrophils pre-incubated with FH from different patients or controls, respectively. Experiments were independently repeated three times. **(F)** Compared with neutrophils pre-incubated with FH from healthy controls, the level of ET-1 in the coculture supernatants was significantly higher when neutrophils were pretreated with FH from patients with active AAV.

One salient feature of AAV is massive endothelial injury, especially glomerular endothelial cells. It is mainly mediated by endothelium–neutrophil interactions and activation of neutrophils on endothelial cells by ANCA (9). Therefore, to assess whether enhanced adhesion mediated by FH would lead to functional effects on neutrophils, the generation of ROS, and the release of lactoferrin were measured in the coculture system of neutrophils and hGEnCs. However, we found that compared with neutrophils pre-incubated with buffer or HSA, FH-treated neutrophils showed less production of ROS (21.6 × 10^4^ ± 3.0 × 10^4^ vs. 30.1 × 10^4^ ± 1.1 × 10^4^, *P* < 0.001; 21.6 × 10^4^ ± 3.0 × 10^4^ vs. 29.2 × 10^4^ ± 3.2 × 10^4^, *P* < 0.001, respectively) (Figure 5A), as represented by the FI of neutrophils cocultured with endothelial cells in each well of the black transparent-bottom plates. Consistently, the level of lactoferrin in the coculture supernatants was also decreased by pre-incubation of neutrophils with FH (2,933.6 ± 157.6 vs. 4,756.3 ± 180.7, *P* < 0.001; 2,933.6 ± 157.6 vs. 4,181.0 ± 368.2, *P* < 0.001, respectively), as compared with pre-incubation with buffer or HSA (Figure 5B). To assess whether the inhibition of neutrophil activation by FH also resulted in subsequent protection of endothelium injury, the level of ET-1, a biomarker of endothelial cell activation and injury, in the coculture supernatants was measured. It was found that FH bound on neutrophils obviously protected against endothelium injury induced by ANCA-mediated activation of neutrophils, whereas the control protein HSA had no effect (24.5 ± 1.5 vs. 35.1 ± 1.5, *P* < 0.001) (Figure 5C). However, we found that compared with FH from healthy controls, FH from patients with active AAV exhibited a deficient ability in inhibiting ANCA-induced neutrophil activation on endothelial cells, including ROS production and degranulation of lactoferrin (30.6 × 10^4^ ± 5.6 × 10^4^ vs. 20.1 × 10^4^ ± 2.0 × 10^4^, *P* = 0.003; 5,017.7 ± 779.0 vs. 3,171.5 ± 164.3, *P* < 0.001, respectively) (Figures 5D,E). In line with this finding, when neutrophils were pre-incubated with FH from patients with active AAV, the concentration of ET-1 in the coculture supernatants was significantly higher than those pre-incubated with FH from healthy controls (37.6 ± 6.4 vs. 20.8 ± 3.9, *P* < 0.001) (Figure 5F), indicating a deficient ability in protecting against endothelium injury mediated by ANCA-induced neutrophil activation. Collectively, these data indicates that FH is capable of inhibiting ANCA-induced neutrophil activation on endothelial cells and protecting against endothelial injury by interacting with neutrophils. However, FH from patients with active AAV exhibited a deficient protective ability.

### Identification of FH Modifications by Mass Spectrometry

**Figure 6:**
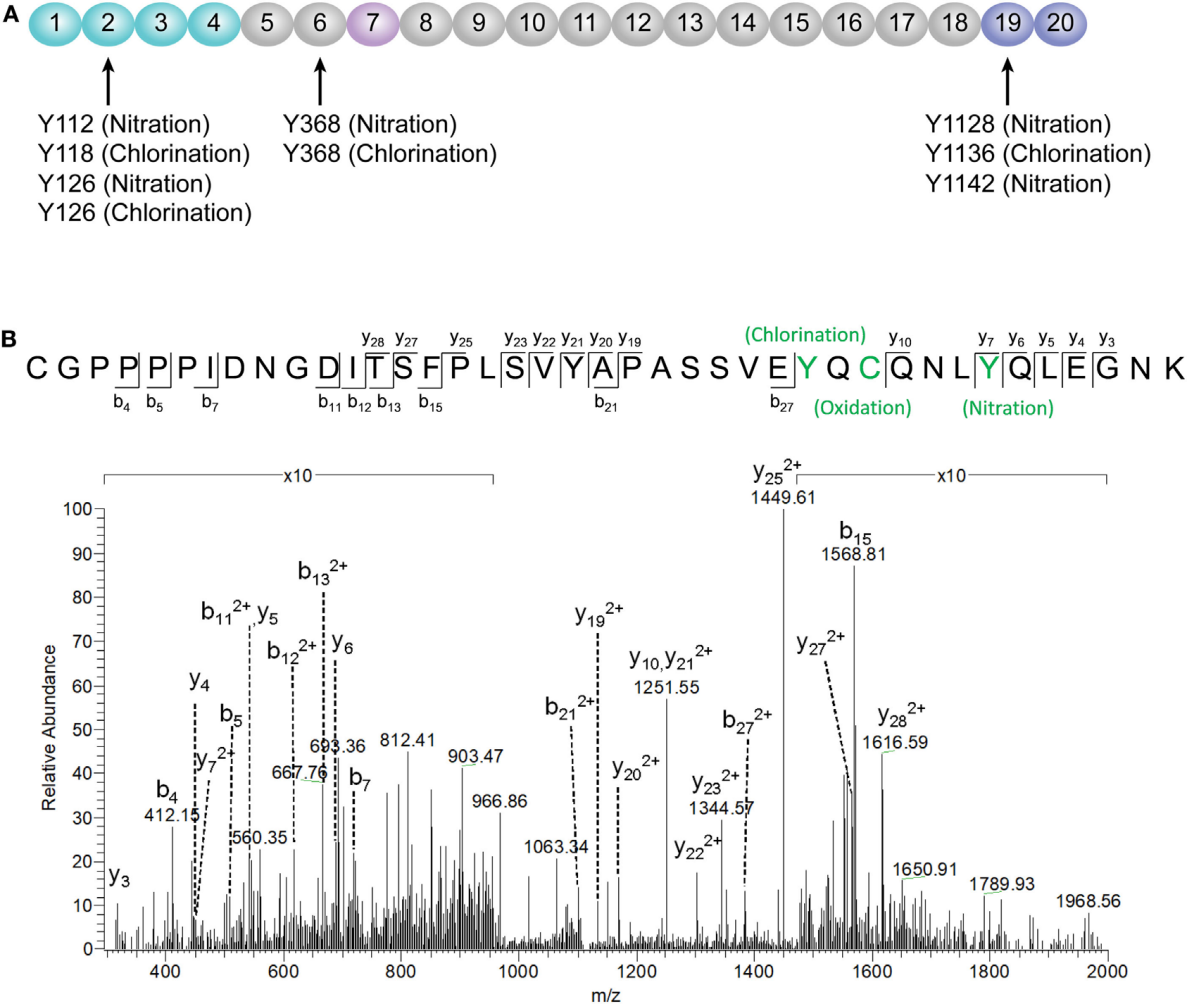
Identification of factor H (FH) modifications by mass spectrometry analysis. **(A)** Summary of all tyrosine nitration and chlorination sites of FH identified in patients with active ANCA-associated vasculitis. The position of the nitrated/chlorinated tyrosine is determined based on the theoretical nitrated/chlorinated peptide tandem mass spectrum with the highest Mascot score. The amino acid sequence of FH is referred to the UniProt database. **(B)** A representative mass spectrogram showing the detection of chlorination of Y1136 and nitration of Y1142 located at SCR19 of FH.

According to a recent published study, tyrosine nitration of FH, an oxidative posttranslational modification, potentiates the activation of monocytes that stimulated with lipid peroxidation by-products (35). Therefore, mass spectrometry was further performed to detect possible modifications of FH in patients with active AAV. Since nitration and chlorination of tyrosine residues could be induced by the MPO H_2_O_2_/Cl^−^ system during inflammatory processes (36), tyrosine nitration and chlorination were searched against the database. The position of the nitrated/chlorinated tyrosine was determined based on the theoretical nitrated/chlorinated peptide tandem mass spectrum with the highest Mascot score (35). A total of nine modification sites were specifically detected in six patients with active AAV as follows: four modifications at SCR2 of FH including two sites of tyrosine nitration and two tyrosine chlorination sites; two modifications at SCR6 including one site of tyrosine nitration and one tyrosine chlorination sites; and three modifications at SCR19 including two sites of tyrosine nitration and one tyrosine chlorination sites. The tyrosine residues of FH that are nitrated or chlorinated detected in patients with active AAV are summarized in Figure 6A. A piece of example tandem mass spectrum showing the nitration and chlorination of FH tyrosine residues is shown in Figure 6B. The result of FH modifications for each patient is listed in Table 1. None of the tyrosine nitration and chlorination sites was detected in FH from all the healthy controls.

**Table 1:** Modification sites of factor H in patients with active ANCA-associated vasculitis detected by mass spectrometry.

P1	P2	P3	P4	P5	P6	P7	P8	P9	P10	P11	P12
Y1136 (chlorination)	None	None	Y368 (chlorination)	None	None	Y1128 (nitration)	None	Y112 (nitration)	None	Y368 (nitration)	Y118 (chlorination)

Y1142 (nitration)						Y1136 (chlorination)		Y126 (chlorination)		Y1136 (chlorination)	Y126 (nitration)

										Y1142 (nitration)	Y368 (chlorination)

## Discussion

Anti-neutrophil cytoplasmic autoantibody-induced neutrophil activation plays a crucial role in the development of AAV (3–8). Our current study showed that FH was capable of inhibiting respiratory burst and degranulation of neutrophils stimulated by ANCA. It extends our previous finding of FH in AAV (20, 21) and indicates a novel role of FH in the pathogenesis of AAV. A number of previous studies demonstrated that CR3 (α_Mβ2_ integrin; CD11b/CD18) serves as a specific receptor for FH on neutrophils (24–26). The α_Mβ2_ integrin has long been implicated in mediating interactions between neutrophils and various types of pathogens (37–40), as well as endothelial cells (28). Losse et al. showed that FH supported attachment and migration of neutrophils to *C. albicans* by interacting with the α_Mβ2_ integrin on neutrophils (25). Consistent with this finding, our current study showed that FH enhanced neutrophil adhesion and migration toward endothelial cells. This result provides evidence to support the hypothesis that FH may facilitate the neutrophils–endothelium interaction by serving as a bridge between neutrophils and endothelial cells (24). Nevertheless, α_Mβ2_ integrin plays a critical role in the process of ANCA-induced activation of neutrophils (9). Interaction of α_Mβ2_ integrin with ICAM-3 expressed on adjacent neutrophils would increase homotypic aggregation of circulating neutrophils (41). And, interaction between α_Mβ2_ integrin and adhesion molecules on endothelial cells such as ICAM-1 facilitates activation of neutrophils in the presence of ANCA including respiratory burst and degranulation, and a substantial increase of ANCA antigen expression (9, 42). Moreover, engagement of α_Mβ2_ integrin together with TNF-α, triggers the clustering of FcγII receptors and the association with NADPH-oxidase components, which is crucial for ANCA-induced activation of neutrophils (27). Neutrophil activation induced by anti-PR3 or anti-MPO antibodies was strongly prevented by blocking α_Mβ2_ integrin (27). Therefore, we speculate that the current observation that FH inhibited ANCA-induced activation of neutrophils may result from the binding of FH to neutrophil α_Mβ2_ integrin. Ligation of FH on α_Mβ2_ integrin may obstruct some important downstream events of it in activating neutrophils. This hypothesis is supported by our findings that FH alone do not activate CR3 (α_Mβ2_ integrin; CD11b/CD18) on neutrophils, nor trigger activation of neutrophils. Consistent with our findings, FH was recently found to be capable of inhibiting phorbol 12-myristate 13-acetate induced release of neutrophil extracellular traps and ROS by neutrophils (26). However, Losse et al. demonstrated that when exposed to *C. albicans* yeasts, FH enhanced the generation of ROS and the release of the lactoferrin by human neutrophils, resulting in a more efficient killing of the fungal (25). It indicates that the effects of FH on neutrophil activation may be differential in different diseases. It also implicates that the FH effect may not be completely explained by the masking effect alone. Besides neutrophils, a recent study by Olivar et al. demonstrated that FH “is able to promote a distinctive tolerogenic and anti-inflammatory profile on monocyte-derived dendritic cells (MoDCs) challenged by a pro-inflammatory stimulus” (43). The anti-inflammatory effect of FH on monocyte/macrophage has also been reported previously (22, 35), while recently Calippe et al. reported a novel role of FH on mononuclear phagocytes (44). The authors showed that FH binding to mononuclear phagocytes inhibits the CD47-mediated homeostatic elimination of mononuclear phagocytes, resulting in non-resolving inflammation within the sub-retinal space, which may be involved in the pathogenesis of age-related macular degeneration (AMD). Collectively, these findings indicate that the effects of FH on different types of cells and in different diseases differ from each other. More importantly, it indicates that therapeutic manipulation of FH requires rigorous disease-specific target validation.

**Figure 7:**
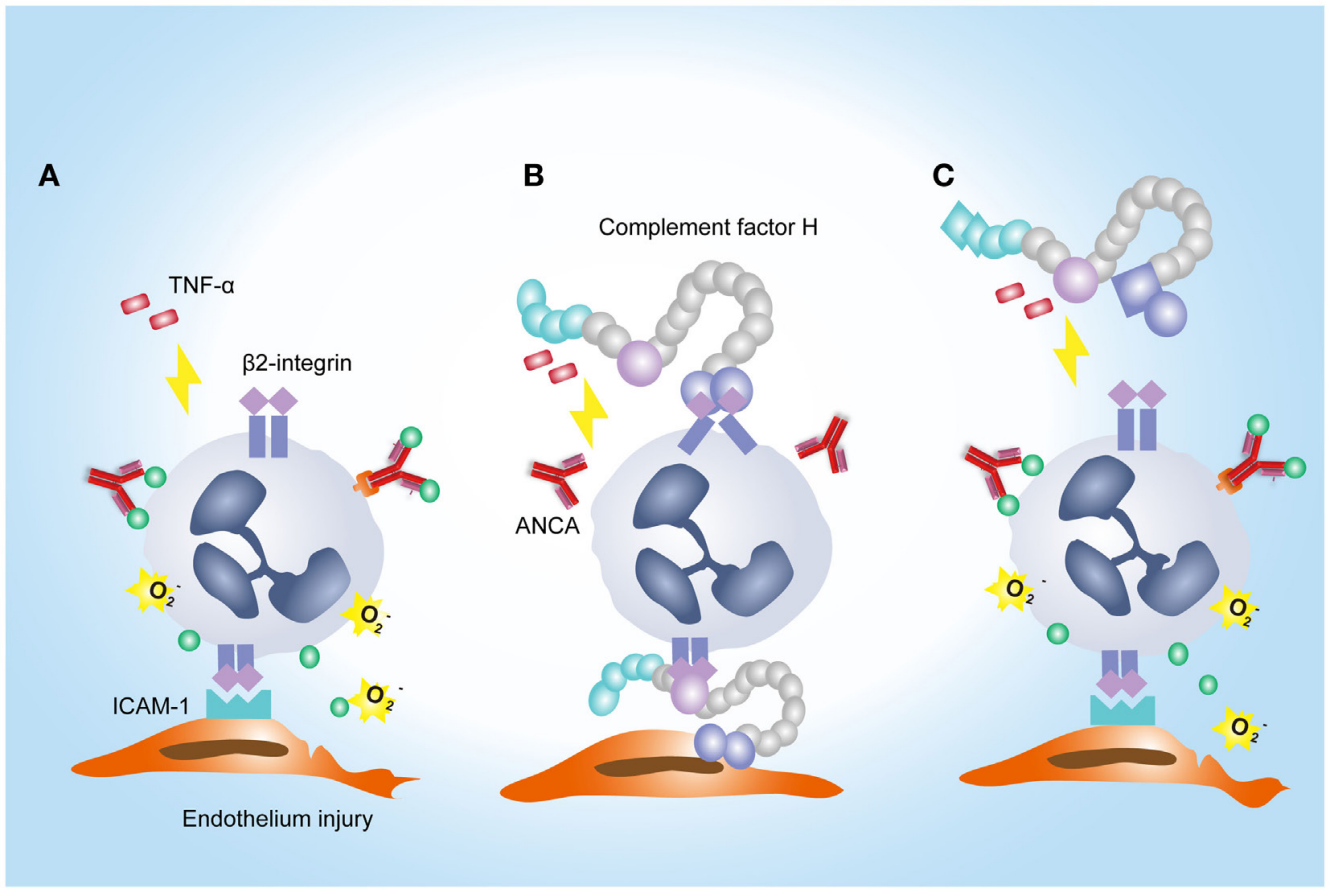
A proposed model of factor H (FH)–neutrophil interaction in the pathogenesis of anti-neutrophil cytoplasmic autoantibody (ANCA)-associated vasculitis (AAV). **(A)** TNF/β2-integrin joint signals triggers explosive responses of neutrophils adherent to endothelial cells in the presence of ANCA, resulting in degranulation and oxidative burst of neutrophils. **(B)** Binding of FH on neutrophils through β2-integrin inhibits ANCA-induced activation of neutrophils. **(C)** In AAV, FH is deficient in binding neutrophils and inhibiting neutrophil activation by ANCA, which may be partly related to tyrosine nitration and chlorination of FH and genetic variations of FH.

Another interesting finding in the current study is that FH from patients with active AAV exhibited a deficient ability in inhibiting ANCA-induced neutrophil activation in fluid phase and on endothelial cells. It potentially implicates an underlying mechanism for the overwhelming activation of neutrophils and destructive endothelium injury in AAV patients. Furthermore, excessive activation of neutrophils would result in more release of factors capable of activating the alternative complement pathway (12, 45, 46). In particular, according to our recent finding, MPO that released from neutrophils upon activation by ANCA, binds to FH and inhibits the complement regulatory activity of FH (47). Therefore, it indicates that FH deficiency may amplify the feedback loop between activation of neutrophils and the alternative complement pathway, thus contributing to the development of AAV. Interestingly, a recent study demonstrated that tyrosine nitration of FH, a type of oxidative posttranslational modification, occurred in patients with AMD (35). According to their finding, nitrated FH not only lost its cofactor activity for factor I-mediated cleavage of C3b but also significantly potentiated the activation of monocytes that stimulated with lipid peroxidation by-products (35). In the context of AAV, ANCA is capable of inducing the release of nitric oxide from human neutrophils (48). By reacting with superoxide, nitric oxide yields the production of oxidant peroxynitrite, an important mediator of nitration (49). Moreover, during inflammatory processes, nitration and chlorination of tyrosine residues could be induced by the MPO/H_2_O_2_/Cl^−^ system (36). In our current study, several tyrosine nitration and chlorination sites were identified in some patients with AAV, and most modification sites were located at SCR1–4 and SCR19–20 of FH. Interestingly, none of the tyrosine nitration and chlorination site was detected in FH from healthy controls and the two patients (patient 8 and 10) who exhibited relatively similar inhibitory effect on neutrophil activation to that from healthy controls. It implicates that nitration and chlorination of FH tyrosine residues may be relevant to the deficient activity of FH in binding neutrophils and inhibiting neutrophil activation, especially given that a main binding site for neutrophils within FH are located in SCR19–20, and a minor site may be present within SCR1–4 (25). In addition, we recently reported several genetic variations of FH in these patients with AAV, including the nonsynonymous variants Val62Ile and Tyr402His (21). The Val62 variant within the SCR1 domain of FH, which was associated with the risk of developing AMD and dense deposit disease (50, 51), showed less effective binding affinity to C3b and cofactor activity of FH compared with FH-Ile62 in previous studies (52–54). Whether it influences the interaction between FH and neutrophils is not clear. But considering that SCR1–4 of FH may serve as a binding site for neutrophils (25), we suspect that the Val62 of CFH might be in part related to the FH functional deficiency in inhibiting neutrophil activation. The Tyr402His variant of FH was found to be strongly associated the risk of AMD (55–57). Recently Calippe et al. showed that the risk variant His402 inhibited the homeostatic elimination of microglial cell, which may be related to a more effectively binding to CD11b, as compared with the non-risk variant Tyr402 (44). Whether it influences the binding of FH on neutrophils has not been elucidated. Limited by a relative small sample size, we are not able to find any firm association of this variant with the binding of FH to neutrophils; especially considering that FH used in the study were all purified from plasma, any modification that occurred in human blood may alter the results. Altogether, we suggest that the mechanism underlying FH deficiency in inhibiting neutrophil activation in AAV patients may be multifactorial. A proposed model of FH–neutrophil interaction in the pathogenesis of AAV has been illustrated in Figure 7. Nevertheless, these findings suggest a potential therapeutic role for FH in AAV, especially given that we recently found that the complement regulatory activity of FH is impaired in AAV patients (21).

In conclusion, FH inhibits ANCA-induced neutrophil activation and protects against glomerular endothelial injury by interacting with neutrophils. However, FH from patients with active AAV is deficient in the ability to bind neutrophils and inhibit neutrophil activation by ANCA. The current findings further extend the understanding of the pathogenesis of AAV, thus providing potential clues for intervention strategies.

## Ethics Statement

Informed consent from each participant was obtained. The study was conducted in line with the Declaration of Helsinki and was approved by the ethics committees of Peking University First Hospital.

## Author Contributions

S-FC conducted the experiments, analyzed the data, and drafted the manuscript. F-MW contributed a part of methods used in the study. Z-YL provided reagents/materials/analysis tools and helped to revise the manuscript. FY participated in the design and guidance of the study and helped to revise the manuscript. M-HZ involved in its design, assisted with interpretation of data, and provided suggestion for revising the manuscript. MC conceived of the study, participated in the revision of the manuscript, and provided final approval of the version of the submitted manuscript. All authors read and approved the manuscript.

## Conflict of Interest Statement

The authors declare that the research was conducted in the absence of any commercial or financial relationships that could be construed as a potential conflict of interest.
