# Supplementary figures and images for: Complement Factor H Inhibits Anti-Neutrophil Cytoplasmic Autoantibody-Induced Neutrophil Activation by Interacting With Neutrophils
Source: Front Immunol. 2018 Mar 19;9:559. doi: 10.3389/fimmu.2018.00559 (PMC5867335; doi:10.3389/fimmu.2018.00559)

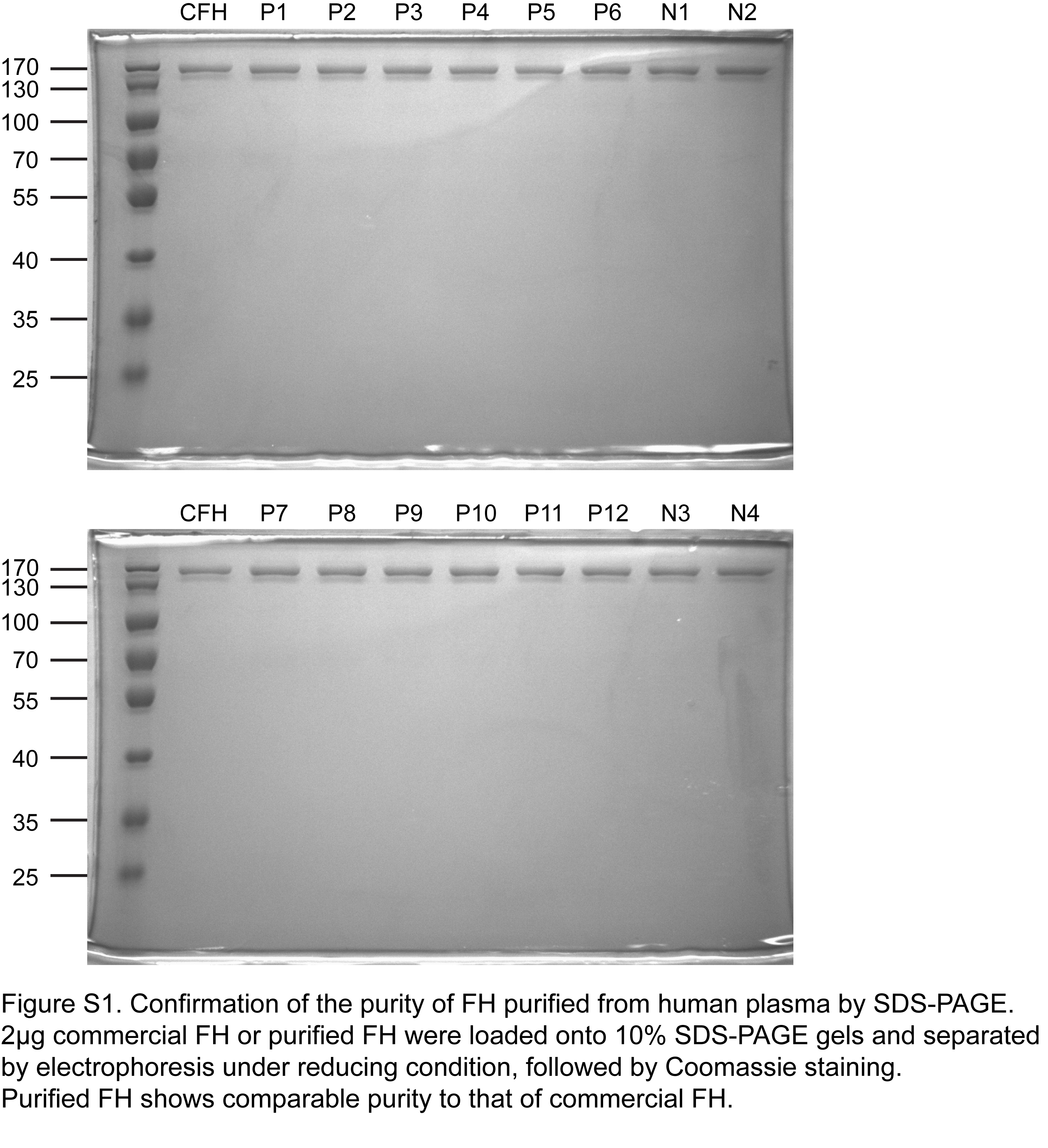

Supplement: Supplementary file 1 [file Image_1.TIF]

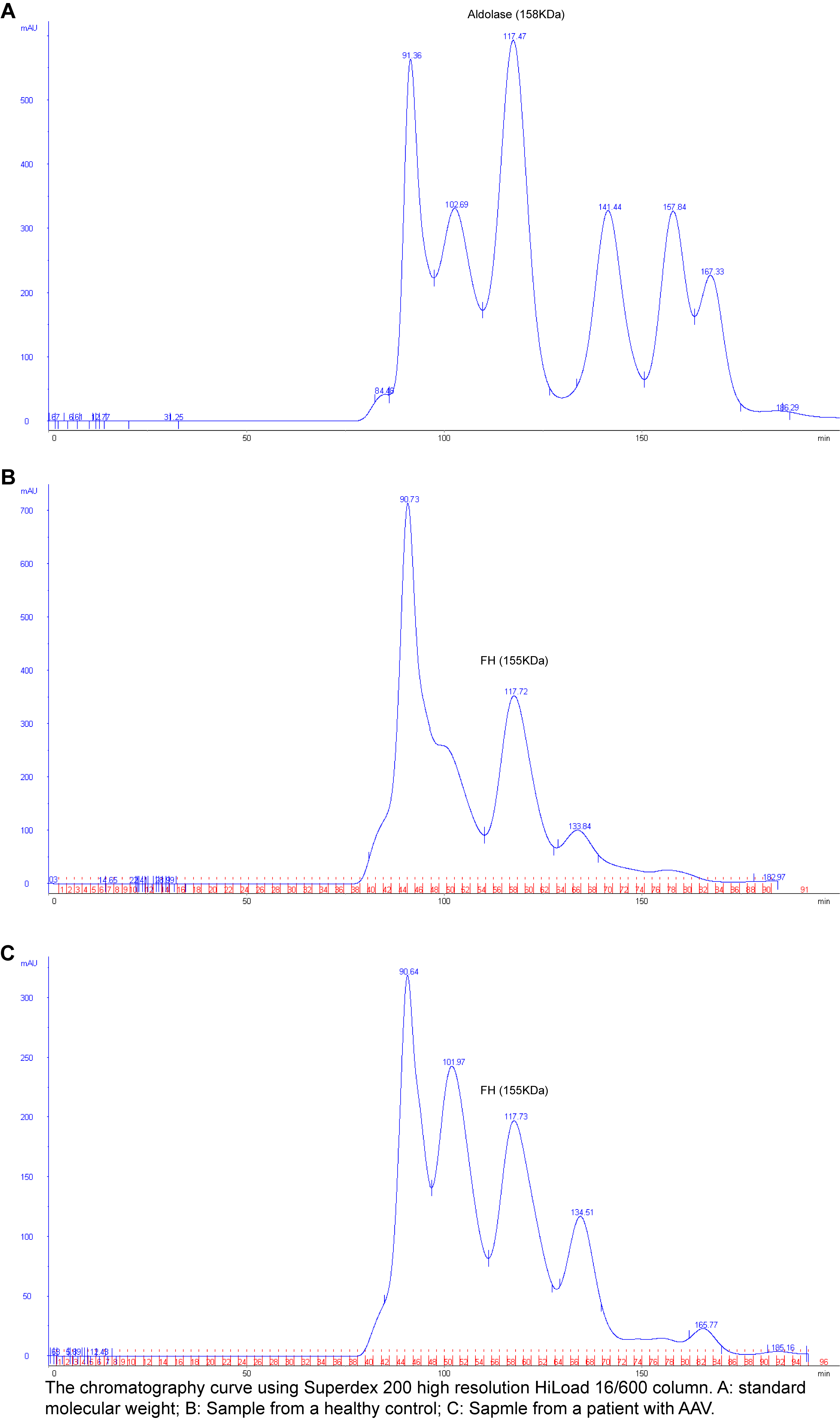

Supplement: Supplementary file 2 [file Image_2.TIF]

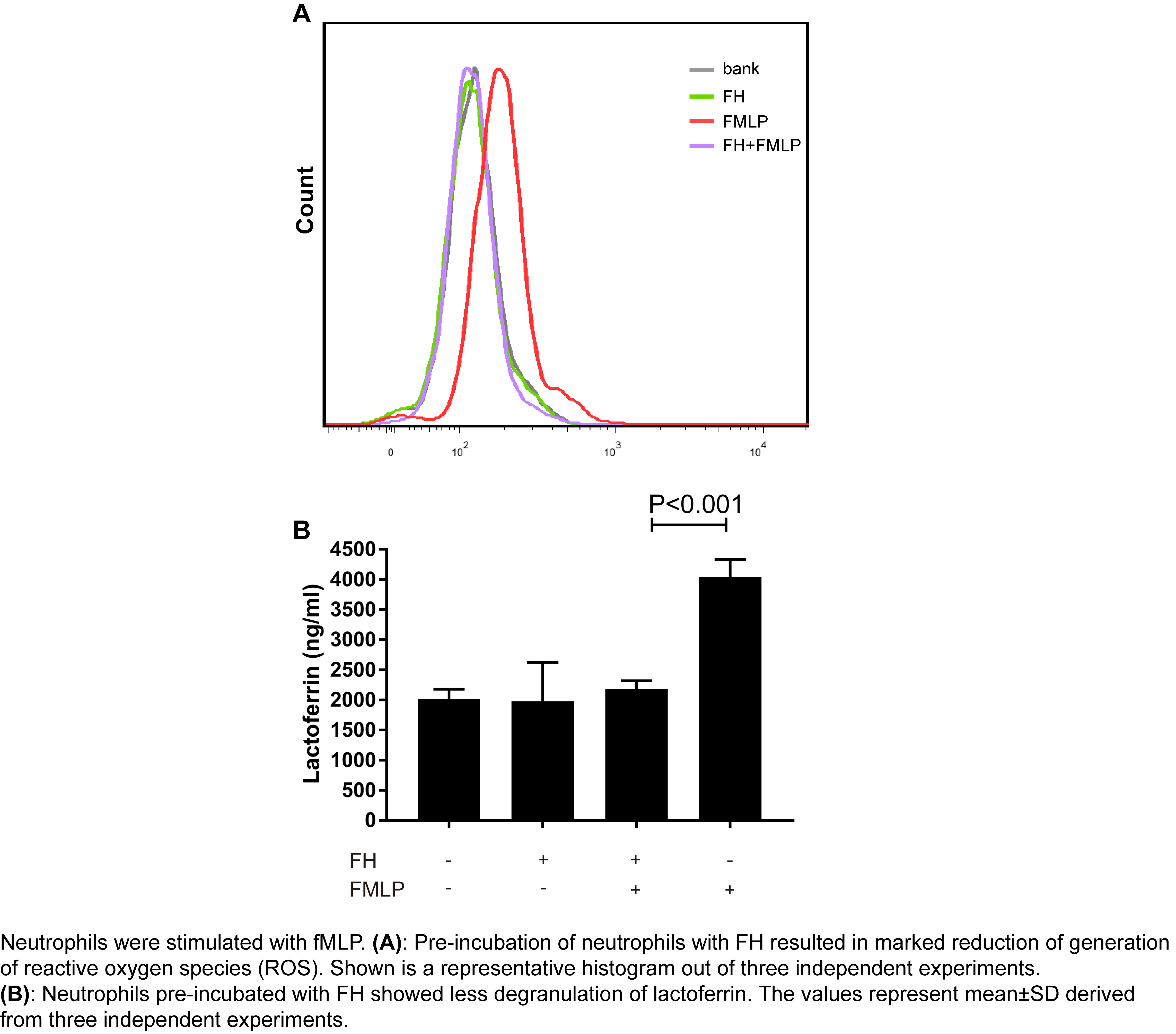

Supplement: Supplementary file 3 [file Image_3.TIF]
